# Conducting national burden of disease studies in small countries in Europe– a feasible challenge?

**DOI:** 10.1186/s13690-021-00599-z

**Published:** 2021-05-11

**Authors:** Sarah Cuschieri, Elena Pallari, Natasa Terzic, Ala’a Alkerwi, Rannveig Sigurvinsdottir, Inga Dora Sigfusdottir, Brecht Devleesschauwer

**Affiliations:** 1 Department of Anatomy, Faculty of Medicine and Surgery, University of Malta, Msida, Malta; 2 MRC Clinical Trials and Methodology Unit, University College London, 90 High Holborn, London, WC1V 6LJ England; 3 Center for Health System Development, Institute of Public Health of Montenegro, Podgorica, Montenegro; 4 Directorate of Health, Service Epidemiologiy and Statistics, Luxembourg, Luxembourg; 5 Department of Psychology, Reykjavik University, Reykjavik, Iceland; 6 Teacher’s College, Columbia University, New York, NY USA; 7 Department of Epidemiology and Public Health, Sciensano, Brussels, Belgium; 8 Department of Veterinary Public Health and Food Safety, Ghent University, Merelbeke, Belgium

**Keywords:** Burden of disease, DALY, YLD, YLL, Morbidity, Mortality, Cyprus, Iceland, Luxembourg, Malta, Montenegro, European Burden of Disease Network

## Abstract

**Background:**

Burden of Disease (BoD) studies use disability-adjusted life years (DALYs) as a population health metric to quantify the years of life lost due to morbidity and premature mortality for diseases, injuries and risk factors occurring in a region or a country. Small countries usually face a number of challenges to conduct epidemiological studies, such as national BoD studies, due to the lack of specific expertise and resources or absence of adequate data. Considering Europe’s small countries of Cyprus, Iceland, Luxembourg, Malta and Montenegro, the aim was to assess whether the various national data sources identified are appropriate to perform national BoD studies.

**Main body:**

The five small countries have a well-established mortality registers following the ICD10 classification, which makes calculation of years of life lost (YLL) feasible. A number of health information data sources were identified in each country, which can provide prevalence data for the calculation of years lived with disability (YLD) for various conditions. These sources include disease-specific registers, hospital discharge data, primary health care data and epidemiological studies, provided by different organisations such as health directorates, institutes of public health, statistical offices and other bodies. Hence, DALYs can be estimated at a national level through the combination of the YLL and YLD information.

On the other hand, small countries face unique challenges such as difficulty to ensure sample representativeness, variations in prevalence estimates especially for rarer diseases, existence of a substantial proportion of non-residents affiliated to healthcare systems and potential exclusion from some European or international initiatives.

Recently established BoD networks may provide a platform for small countries to share experiences, expertise, and engage with countries and institutions that have long-standing experience with BoD assessment.

**Conclusion:**

Apart from mortality registries, adequate health data sources, notably for cancer, are potentially available at the small states to perform national BoD studies. Investing in sharing expert knowledge through engagement of researchers in BoD networks can enable the conduct of country specific BoD studies and the establishment of more accurate DALYs estimates. Such estimates can enable local policymakers to reflect on the relative burden of the different conditions that are contributing to morbidity and mortality at a country level.

## Background

Burden of Disease (BoD) studies calculate disability-adjusted life years (DALYs) to quantify the health of a population through a single metric. DALYs are calculated through the combination of ‘years lived with disability (YLD)’ and ‘years of life lost (YLL)’ [1]. The YLD calculation relies on prevalence data. As part of the YLD calculation, disability weights (DWs) are required. The DWs depend on the population perception of living with a particular condition for a period of a year. Disability weights ranges from 0, equivalent to optimal health, to 1, equivalent to death. These DWs tend to have a high level of agreement across different populations for the majority of the conditions. Therefore, small countries may consider using the already established DWs by the GBD study [2]. Using DWs enable us to compare between a year lost to death and a year spent with a specific disease, injury or risk factor [1]. YLDs furthermore require severity distributions of conditions (e.g. mild/moderate/severe), which, in contrast to DWs, cannot be considered to be identical across countries. Indeed, it was reported that consideration for the development of country specific severity distributions is merited [3]. However, considering the scarce resources, such an exercise would be challenging, especially for small countries.

BoD estimates provide the foundations for planning and prioritizing health policies at a population level, as well as evaluating the effectiveness of interventions. Hence, it is essential that such metric is regional- or country-specific. The Global Burden of Disease (GBD) study provides estimates of BoD for regions and countries across the world and is based on a comprehensive methodology of complex statistical models and specific assumptions [4]. However, concerns have been raised about the methodology and the accuracy of the severity distributions used in the GBD study, with the recommendation to develop estimates on country-specific data [3, 5]. The aim of this manuscript was to explore whether small countries have conducted national BoD studies and whether their available data sources are appropriate for conducting country-specific BoD studies.

## Europe’s small countries

The small countries considered in this work are Cyprus, Iceland, Luxembourg, Malta and Montenegro, all of which are participating members of the COST Action “European burden of disease network” [6]. The GBD 2019 study provides BoD estimates for these countries. However the data sources used for these estimations are not all locally-based studies or sources [2].

## Availability of data sources

### Mortality data

All considered European small countries have a central ICD10-based mortality registry or database managed by the Directorate of Health or Public Health authorities of the respective country. Death certificates are filled in by trained doctors and each certificate is inputted by a trained coder. In some countries, such as in Iceland, death certificates allow for multiple causes of death and hence multiple ICD10 codes are required. In this case, the inputting of the death certificate is reviewed by a second trained coder, a doctor, to ensure the entrance of adequate and reliable ICD-10 codes. Similarly, in Luxembourg, more than one coder is responsible for inputting death certificates. This typically occurs when technical issues are flagged on inputting such as unreadable handwriting, handling missing data and in the case of an external cause of death. This process is followed to ascertain reliability in the inputting of the death certificate within the registry. Although, such inputting strategy will limit ill-defined causes of deaths (IDD). However, Montenegro face challenges with IDD due to their recent shift in the responsible authorities for the mortality database system, therefore, the registry is currently not yet fully functional. Hence, calculation of YLLs following the GBD methodology [7] is feasible, but the accuracy of the estimation is country-dependent.

### Morbidity data

A number of health information data sources can be used to monitor the prevalence and severity distributions of diseases, injuries or risk factors. The feasibility to use the already published national data as a source to calculate the YLD is dependent on the country’s population coverage and quality of data. It is also dependent on a whether these national data sources provide information on disease prevalence and its severity.

#### Health information sources

Overall, each small country has an entity that records health information at the population level, such as a Health Directorate, an Institute of Public Health or Statistical Office. Such health information system is representative of the population and suitable for estimating prevalence of diseases, risk and injuries. A question lies as to the ease of accessibility to all the data and the integration of such data for YLDs calculation, especially since severity of the condition may not be readily available from  a single data source and would depend on other sources. In the digital health era, linking data may be feasible if a common identifiable code/number is assigned to each patient, enabling cross-linkage of data across the data platforms. Researchers can use such data sources provided that appropriate permissions were gathered before or during data collection to allow for anonymised data linkage and use. This requires the presence of secured health information infrastructure, which may not be available, especially in small countries. Small countries typically have limited resources and are more likely to invest in the set-up of healthcare service infrastructure rather than the collection and linking of health information data [8].

Primary health care (PHC) data can also provide invaluable information. In Montenegro, the PHC data has been integrated with the Institute of Public Health database with the goal to enable analysis for research purposes. Such a data linkage may provide an adequate data source for BoD studies. However, not all small countries can access such data freely, whilst others may be faced with a methodological challenge of establishing sample representativeness of the population using such data. In Malta, although the PHC is state run and free for all tax-paying residents, only a proportion of the population seek medical advice and care at PHC. The majority of the population opts to visit private general practitioners that do not have a common data sharing platform, making this data source unreliable for BoD studies.

#### Disease-specific registries

The availability of different registries among the different small countries might help to estimate the prevalence data required for certain conditions. Table 1 gives a comparative summary of the registries available for each small country. A number of different disease registries are available within these small countries. The common ones are cancer registries.

**Table 1:** Comparative summary of the different disease-specific registers available in the different small European member states

Morbidity registry	Cyprus	Iceland	Luxembourg	Malta	Montenegro
Cancer	Yes	Yes	Yes	Yes	Yes
HIV/AIDS	Yes	No	Yes	No	No
Communicable diseases	No	Yes	Yes	No	Yes
Diabetes	Yes	No	No	No	Yes
Cardiovascular disease	No	Yes	No	No	Yes
Nosocomial infections	No	No	Yes	No	No
Perinatal health	No	No	Yes	No	No
Rare diseases	No	No	No	Yes	No
Cerebral palsy	No	No	No	Yes	No
Birth defects	No	No	No	Yes	No
Dementia	No	No	No	Yes	No
Hospital discharge/statistics	No	Yes	Yes	Yes	No
Prescription medicines	No	Yes	No	Yes	No
Drug and drugs addiction	No	No	Yes	No	YEs
Trauma and accident	No	Yes	Yes	No	No
Vaccination	No	Yes	No	Yes	Yes
Transplants	No	No	No	Yes	No

In Malta, different registries are being kept by different entities. An example is the population free medicine prescription registry for chronic diseases. This is kept by the ‘Pharmacy of Your Choice (POYC)’ unit which falls under the umbrella of the Ministry for Health, whilst the cancer registry is kept by the Directorate of Health Information and Research. A similar scenario is present in Luxembourg, where different entities are responsible for the management of different registries. This is a clear example of fragmentation of data sources. Using disease-specific registries as a source for YLDs calculations comes with advantages and disadvantages. An advantage is that medical professionals are usually responsible for the reporting of cases using standardised protocols which are then recorded within the registers. This data source may also be appropriate for disease severity distribution estimations. Compared to data available from epidemiological studies, such registers, may not include all patients falling within the register’s category, due to discrepancies in the categorisation of patients occurring from interpersonal bias. This may also result in missing out patients, as individuals with a particular disease, may be opting out of visiting the healthcare system and hence may  not be registered within the database. Such population representation of the data source is dependent on the country population attitudes and behaviours with respect to the use of healthcare services.

#### Hospital discharge data

Hospital discharge data is another potential source that can be used by small countries for BoD studies, which provides information on prevalence and severity distribution of diseases. Of note, this kind of data will only be able to account for the upper end of the disease’s severity scale that require admission. This may have an effect on the prevalence estimations of certain diseases and injuries.

Hospital discharge data is available across all the public hospitals of the small countries under study, although accessibility and quality of this data differs by country. For example, in Malta, the digital hospital discharge data is only available from the year 2007 onwards. Discharge data documentation is the responsibility of the ward doctor and details provided as part of this report is subject to the ward doctor’s writing preference.

An additional issue in utilization of hospital discharge data is the degree of population data representation. This is especially true for the countries that have a number of public and private hospitals. The reliability of hospital discharge data will hence vary between countries. For example, in Iceland where there are only public hospitals, hospital discharge data may be a reliable morbidity data source for BoD studies. Montenegro also has only public hospitals, therefore, hospital discharge data are nationally representative. However, tracking data by patient imposes an issue, as data are mostly available in an aggregated form. In countries where a mixture of public and private hospitals is present (such as in Malta), using hospital discharge data provided by public hospitals may not be a representative yield of the whole country. The case for Cyprus is different as it has recently undergone the implementation of a general health system serving as the aegis for the direct competition of public and private hospital in universal coverage provision [9]. The responsible unit for such data remains the Monitoring Health Unit (MHU). Similarly, in Luxembourg, a new hospital documentation system (www.dcsh.lu) is being set up that combines the medical documentation of all public and private hospitals together through diagnosis-related groups (DRG) using two classifications for diagnosis (ICD-10 CM) and for procedures (ICD-10 PCS).

#### Health insurance data

Private health insurance is not obligatory for the population of these small countries since health care is provided as part of the National Health Insurance. Therefore, using private health insurance data may not be representative of the small country’s characteristics and should not be considered as the primary morbidity data source.

#### Epidemiological studies

A number of epidemiological studies have been conducted in these small countries. These studies fall within the frame of the ‘European Health Interview Surveys (EHIS)’, which follow the European Commission Regulation for health statistics. The corresponding datasets are easily accessible through Eurostat and provide information on the health status, health care use, health determinants and socio-economic background of each country (https://ec.europa.eu/eurostat/data/database). A number of other nationally representative surveys have been carried out in Iceland, Luxembourg, Malta and Montenegro, focusing on different aspects of health in adults and in children including specific age groups (such as the Health Behaviour in School-age Children – HBSC) and others focused on non-communicable disease risk factors (tobacco use and alcohol consumption, obesity). Surveys conducted in small countries are typically representative of the population and may also be a good source for estimating prevalence and disease specific severity distribution. The challenge however remains whether data from such sources can be used to draw comparisons between countries, since these surveys (especially health examination surveys) are not run on a standardized basis.

### Feasibility and challenges for national burden of disease study in small countries

The small population size may constitute an advantage for the collection of health information; however, these countries are also faced with challenges arising from the lack of resources and expertise in certain areas. Among other barriers, small countries have distinctive challenges related to limited accessibility to health data and information, hampering them from performing national BoD studies.

To date, the small countries in question have not conducted national BoD studies. A number of different health information sources are readily available in each of the small countries that can provide prevalence-based data for calculation of YLDs and mortality data for calculation of YLLs, both of which are required to perform national BoD studies. However, most health data sources are typically fragmented with routine country health statistics and epidemiological studies being conducted separately and kept by different entities. The concern about data fragmentation is that data linking may not always be possible even in small countries, such as data gathered by the state cannot be linked to data gathered by private entities such as insurance companies or general practitioners. Of note, absence of standardization of data capturing and storage among small countries could be clearly observed in Table 1. A difference in the type of disease and injuries registries was evident, which may hinder countries in conducting comparative BoD studies. These issues may be originating from scarce resources that affects the set-up of health data infrastructure. Furthermore, some small countries may face legal and technical constraints against unique data codification and linkage from different sources, which will make data accessibility problematic. Additionally, using multiple sources to calculate YLD may be subject to overestimation of the condition’s impact even more so if multimorbidity is present. Hence, it is essential that data triangulation is performed by following a data linkage process through a unique identifiable number (if available) to ensure a creditable of the data source.

Achieving a survey/study’s population sample representation is another challenge faced by small countries. A representative survey depends on the number of responders. Small countries require a greater proportion of the population to be sampled and to participate to maintain population representation than larger countries [8]. Also, the per capita cost of a survey tends to be larger to run in a small country than a larger one [8]. Hence, considering these challenges along with the fact that small countries have typically limited resources and low participation rates (as experienced by other countries), sample study representation may be a significant issue.

The use of secondary data can lead to inaccuracies such as overestimation of the DALYs, which can be avoided by conducting full BoD studies of the different conditions that affect each country [10]. It is recommended, as a first step, to undergo a pilot BoD study to ensure that the methodology and the outcomes are reliable and comparable to other countries. Indeed, a harmonized methodology is a requisite along with performing of standardization by measuring BoD rates per 100,000 population. However, this requires a vast number of resources and expertise, which are considered as very limited in these small countries. In addition, the concept of BoD may not have been recognised as an essential tool for policymaking in small countries. Hence, this may present an additional hurdle in financing and supporting the conduct of national BoD studies.

Small countries face specific challenges such as the presence of a very small number of cases and/or deaths for a particular condition or rare diseases. This may influence the prevalence estimates in contrast to larger countries where the estimated prevalence would not drastically change with the addition of new diagnosed cases. In order to avoid potential skewing of the results, these cases may require the grouping of conditions into broader categories, which will have an effect on the accuracy of YLD’s and YLL’s measurements. This may give an inaccurate picture of the BoD for small countries.

Another challenge faced by some small countries comes from the relatively large proportion of non-residents within the country. An example is Luxembourg, where a number of ‘cross-border’ employees who work in the country are affiliated with the national health security system. Therefore, using such data to estimate the YLDs would over/under-represent the BoD. In Iceland and in Malta a proportion of the residents do not hold an Icelandic or Maltese citizenship, however, are eligible for social and health services since they originate from the European Economic Area (provided they have been residents for 6 months). Hence, the morbidity data available will incorporate national citizens and immigrant population, which again will have an effect on the DALYs. In theory this could be addressed by performing BoD studies stratified by nationality; however, information on nationality is not available in all health data sources, preventing such an approach. Finally, some small countries (e.g. Iceland and Montenegro), that are not part of the European Union (EU), may not be eligible to participate in certain European or international initiatives.

## The way forward

As the BoD concept gains prominence, an increasing number of countries are exploring the possibility to conduct national BoD studies. In this respect, small states are confronted with a number of challenges – some common to all countries, some very specific to the small size of the population and the public health sector. To help address these challenges, recently established BoD networks may provide a platform for small countries to share experiences and expertise with each other, and to engage with other countries and institutions that have long-standing experience with BoD assessment [6]. Through these networks, small countries may further develop their capacity in BoD assessment and find opportunities for jointly addressing the challenges they and other countries are faced with.

## Conclusions

Small European member states have adequate data sources that can be used for national BoD studies. However, better co-ordination between departments is needed, as well as the use of international BoD expertise and resources. Therefore, it is considered that investing in such expertise can yield more accurate DALYs estimates. Such estimations can enable the local policymakers to identify the population needs more accurately, as well as reflect on the relative burden of the different conditions that are contributing to morbidity and mortality at a country level. Establishing data sources and supporting the conduct of BoD studies can contribute to better research and policymaking in these small states.

## Data Availability

The datasets used and/or analysed during the current study are available from the corresponding author on reasonable requests.

## Abbreviations

BoD: Burden of Disease
DALYs: Disability adjusted life years
DWs: Disability weights
EHIS: European health interview survey
GBD: Global burden of disease
HBSC: Health Behaviour in School-Aged Children
ICD-10: International classification of disease
ICD-10 PCS: Procedure Coding System
ICD 10 CM: Clinical Modification
IDD: Ill-defined cause of death
PHC: Primary health care
SEMS: Small European member states
YLD: Years lived with disability
YLL: Years of life lost

## References

[1] Murray CJ, Lozez AD (1996). The Global burden of disease : a comprehensive assessment of mortality and disability from diseases, injuries, and risk factors in 1990 and projected to 2020 : summary.

[2] Lim SS, Abbafati C, GBD 2019 Diseases and Injuries Collaborators T (2020). Global burden of 369 diseases and injuries in 204 countries and territories, 1990–2019: a systematic analysis for the Global Burden of Disease Study 2019. Lancet.

[3] Wyper GMA, Grant I, Fletcher E, Chalmers N, McCartney G, Stockton DL (2020). Prioritising the development of severity distributions in burden of disease studies for countries in the European region. Arch Public Health.

[4] Murray CJL, Frenk J, Piot P, Mundel T (2013). GBD 2.0: a continuously updated global resource. Lancet.

[5] Yoon S-J, Kim Y-E, Kim E-J (2018). Why they are different: based on the burden of disease research of WHO and Institute for Health Metrics and Evaluation. Biomed Res Int.

[6] Devleesschauwer B (2020). European burden of disease network: strengthening the collaboration. Eur J Pub Health.

[7] Naghavi M, Wang H, Lozano R (2015). Global, regional, and national age-sex specific all-cause and cause-specific mortality for 240 causes of death, 1990-2013: a systematic analysis for the Global Burden of Disease Study 2013. Lancet.

[8] Calleja N, Garthwaite PH (2016). Running an international survey in a small country. Challenges and opportunities. Public Heal Panor.

[9] Pallari E, Samoutis G, Rudd A (2020). Re-engineering the Cypriot healthcare service system. BMC Health Serv Res.

[10] O’Donovan MR, Gapp C, Stein C (2018). Burden of disease studies in the WHO European Region-a mapping exercise. Eur J Pub Health.

